# NMR resonance assignment of the N-terminal GTPase domain of human Miro2 Bound to GTP

**DOI:** 10.1007/s12104-022-10103-5

**Published:** 2022-09-01

**Authors:** Cassandra E. Smith, David N. M. Jones

**Affiliations:** 1 Program in Structural Biology and Biochemistry, University of Colorado School of Medicine, Anschutz Medical Campus, Aurora, CO 80045 USA; 2 Department of Pharmacology, University of Colorado School of Medicine, Anschutz Medical Campus, Aurora, CO 80045 USA

**Keywords:** Miro2 N-terminal GTPase domain, Mitochondria, Solution state nuclear magnetic resonance, Backbone and sidechain nuclear magnetic resonance assignments, Chemical shifts

## Abstract

Miro2 and Miro1 are mitochondrial-associated proteins critical for regulating mitochondrial movement within the cell. Both Miro1 and Miro2 have roles in promoting neuron function, but recently Miro2 has been shown to have additional roles in response to nutrient starvation in tumor cells. Miro1 and 2 consist of two small GTPase domains flanking a pair of EF-hands. The N-terminal GTPase (nGTPase) domain is responsible for initiating mitochondrial trafficking and interactions with GCN1 in prostate cancer. The crystal structure of Miro1 nGTPase bound to GTP has been solved. However, no structural data is available for the nGTPase domain of Miro2. To better understand the similarities and differences in the functions of Miro1 and Miro2, we have initiated structural studies of Miro2. Here we report the backbone NMR chemical shift assignments of a 22 KDa construct of the nGTPase domain of Miro2 bound to GTP that includes residues 1–180 of the full-length protein. We affirm that the overall secondary structure of this complex closely resembles that of Miro1 nGTPase bound to GTP. Minor variations in the overall structures can be attributed to crystal packing interactions in the structure of Miro1. These NMR studies will form the foundation for future work identifying the specific interaction sites between Miro2 and its cellular binding partners.

## Biological context

Miro1 and Miro2 regulate the proper spatial distribution of mitochondria in response to stimuli. The function of these proteins has been most studied in neurons but has also been characterized in other cell types (Desai et al. 2013). Dysfunction of Miro1 in neurons has been linked to neurodegeneration (Panchal and Tiwari 2021), while more recently, Miro2 was found to have additional roles in the progression of prostate cancer (Furnish et al. 2022).

**Fig. 1:**
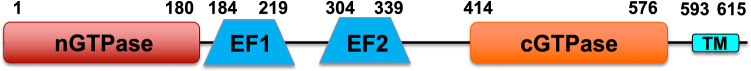
Domain structure of Miro2 Miro2 contains two small GTPase domains at the N- and C termini flanking two EF-hand domains. A transmembrane C-terminal helix anchors the protein to the outer mitochondrial membrane

Miro1 and Miro2 are multidomain proteins containing two small GTPase domains, (nGTPase and cGTPase), flanking two EF-hand domains (Fig. 1) and are constitutively linked to mitochondria through a C-terminal transmembrane domain. Mutation experiments show that the nGTPase domain is critical for initiating mitochondrial trafficking in response to stimuli (Babic et al. 2015). Typically, the release of GDP and subsequent binding of GTP induces a conformational change in small GTPase proteins leading to an increased affinity for an effector protein (Spoerner et al. 2001), eliciting a downstream cellular response (Aspenstrom 1999). Miro nGTPase domains have been classified as atypical (Fransson et al. 2003), creating skepticism about their ability to function like canonical small GTPases. However, mutations that are proposed to mimic the GTP- and GDP-bound conformations display distinct cellular phenotypes (Babic et al. 2015), suggesting that the nGTPase domains have retained canonical function.

The activity and interactions of small GTPases are regulated by five conserved motifs (named G1–G5) that allow the protein to orchestrate conformational changes in response to the binding of GDP or GTP and select for and tightly bind to the guanine base. The function of each of these motifs was first studied in H-Ras (Pai et al. 1989) and has been upheld by subsequent studies of other related canonical small GTPases that have retained all five motifs.

In canonical small GTPases, residues in the rigid G1 loop motif (also referred to as the P loop) directly contact the nucleotide phosphates and mitigate the effect of the additional charges from the phosphates (Saraste et al. 1990) Motifs G4 and G5 provide multiple direct interactions with the guanine base and the ribose to ensure that the nucleotide is bound tightly in the pocket (Pai et al. 1989) and to provide selectivity for guanine (Rensland et al. 1995; Vincent et al. 2007). These regions do not show conformational changes between GDP- and GTP-bound form. In contrast, residues in the G2 motif (also referred to as switch I) and the G3 motif (switch II) differ significantly between the GTP- and GDP-bound conformations (Goody et al. 1992). The GDP-bound form is often highly dynamic (Mello et al. 1997), and the binding of GTP induces a conformational change through contacts with the gamma phosphate (Spoerner et al. 2001; Hall et al. 2002), which creates a stable binding interface for the effector protein (Milburn et al. 1990).

Miro1 and Miro2 nGTPases have retained the G1, G4, and G5 motifs. However, they do not contain canonical G2 and G3 motifs, which should induce a conformational change and mediate effector binding (Eberhardt et al. 2020). Therefore, the exact mechanism by which Miro2 nGTPase receives upstream stimuli to produce the downstream response of mitochondrial trafficking initiation is unknown. Many proteins have been determined to interact directly with Miro nGTPases (Macaskill et al. 2009; Oeding et al. 2018) and recently the interaction of Miro2 with GCN1 was shown to be critical in driving prostate cancer progression. This interaction also occurs through the nGTPase domain as clinically relevant mutations in this domain significantly impact the level of interaction (Furnish et al. 2022).

Our goal is to understand how interactions of Miro2 with its downstream effectors contribute to its function. We aim to identify the specific sites of interaction of different effectors with Miro and how the binding of different nucleotides impacts these interactions. For this, we obtained the backbone assignments of Miro2 nGTPase bound to GTP using a combination of ^13^C/^15^ N and ^2^H/^13^C/^15^ N labeled proteins. We show that the secondary structure closely resembles Miro1 nGTPase-GTP and that observed differences between the Miro1 crystal structure and Miro2 solution structure are most likely a result of packing contacts formed in the crystals of Miro1.

## Methods and experiments

### Protein expression and purification

To obtain GTP-bound nGTPase, residues 1–180 of RhoT2 (Miro2) cDNA (Sinobiological) were PCR amplified and subcloned into the Nde1/XhoI site of the pET28a expression vector, creating an N-terminal His_6_-tag for purification. The plasmid was transformed into *Escherichia coli* BL21 (DE3) competent cells for overexpression. Cultures were grown in Luria broth at 37° C to OD_600_ = 0.6. Cells were harvested at 3000 rpm and resuspended in labeled media for expression. Double labeled (^13^C–^15^ N) protein was expressed in M9 media containing 1 g/L ^15^NH_4_Cl and 2 g/L ^13^C-glucose, and ^2^H–^13^C–^15^ N labeled protein was expressed in M9 media containing 1 g/L ^15^NH_4_Cl and 2 g/L ^13^C-glucose in 99.9% D_2_O. Cultures were grown at 37 °C to OD_600_ = 0.8. Protein expression was induced by adding 0.125 mM isopropyl 1-thio-beta-d-galactopyranoside (IPTG), and cells were grown for 6 h at 18 °C. Cells were harvested by centrifugation at 5000 rpm for 15 min at 4 °C and resuspended in 50 mM HEPES, 500 mM NaCl, 1 mM DTT, 1 mM MgCl_2,_ 5% sucrose, 10 mM imidazole, 0.2 mM GTP, pH 7.5 and lysed by sonication on ice. The lysate was clarified by centrifugation at 15,000 rpm for 20 min. The soluble fraction was loaded onto Ni–NTA column (GE Pharma), equilibrated with 50 mM HEPES, 500 mM NaCl, 1 mM DTT, 1 mM MgCl_2_, 5% sucrose, 10 mM imidazole, pH 7.5. The Ni–NTA resin was washed using a step gradient by increasing the imidazole concentration from 10 to 25 mM and then to 50 mM. The protein was eluted in the same buffer containing 250 mM imidazole and diluted fivefold in 25 mM HEPES 1 mM DTT 1 mM MgCl_2_, pH 7.4. The protein was then loaded onto a HiTrap Q column (GE Pharma) and eluted using a linear gradient against 25 mM HEPES, 1 M NaCl, 1 mM DTT, 1 mM MgCl_2_, pH 7.4. Protein was concentrated and further purified by size exclusion chromatography (Superdex 75, GE Healthcare) with buffer containing 25 mM HEPES, 150 mM NaCl, 1 mM DTT, 1 mM MgCl_2_, 5% sucrose, pH 7.5. Protein purity was assessed by SDS-PAGE to be greater than 95%. Protein concentration was determined by UV absorbance at 280 nm using a NanoDrop (Thermofisher) and a molar extinction coefficient of 20,050 M^−1^ cm^−1^ for a 1:1 complex with GTP.

### NMR spectroscopy

For NMR measurements, the protein was concentrated to 0.3–0.4 mM, and 0.2 mM GTP, 5 mM DTT, and 10% D_2_O (v/v) were added immediately prior to data acquisition. NMR experiments were performed at 25 ˚C on a Bruker Avance Neo 600 MHz equipped with a cryoprobe. Assignments of the protein main-chain atoms were made using sensitivity enhanced versions of 2D ^1^H/^15^ N-HSQC (Kay et al. 1992), 3D HNCO (Grzesiek and Bax 1992b, Muhandiram and Kay 1994), 3D HNCACO (Clubb et al. 1992), 3D HNCACB, CBCA(CO)NH (Grzesiek and Bax 1992a) and (H)CC(CO)NH (Grzesiek et al. 1993) of protonated samples and 3D HN(CO)CA, HN(COCA)CB and HNCACB (Grzesiek and Bax 1992b; Yamazaki et al. 1994) of per-deuterated samples. For per-deuterated samples, no additional procedures were used to back exchange labile protons as cross peak for all resonances were observed in the ^1^H–^15^ N HSQC with comparable intensities as the non-deuterated samples.

All 3D experiments were collected using non-uniform sampling methods (Barna et al. 1987) using the Poisson-gap sampling schemes implemented by Hyberts et al. (Hyberts et al. 2010) and with a sampling density of 35–40%. Data were processed using NMRpipe (Delaglio et al. 1995) and NUS data were reconstructed using SMILE (Hyberts et al. 2012, 2014), and resonance assignments were determined using Ccpnmr Analysis v 2.4.2 (Vranken et al. 2005).

### Extent of assignments and data depositions

**Fig. 2:**
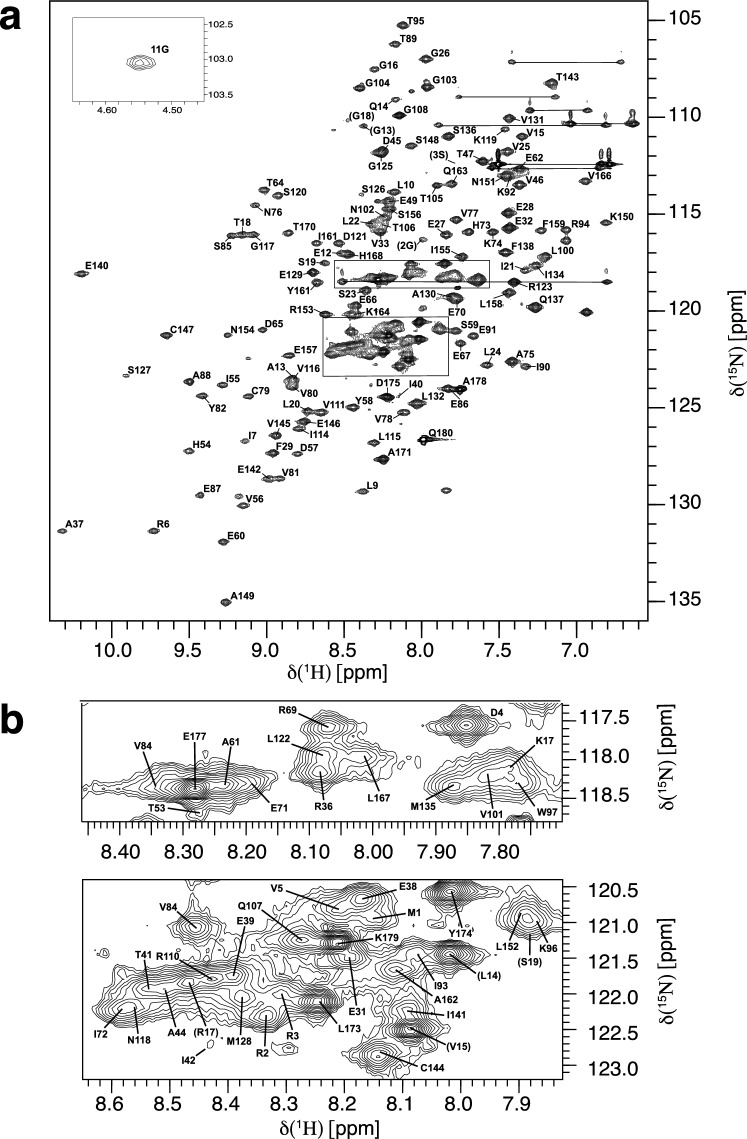
NMR assignments of Miro2 nGTPase **a** Two-dimensional ^1^H–^15^ N HSQC spectrum of ^15^ N/^13^C labeled protein showing the residue assignments of Miro2 nGTPase-GTP complex, The inlaid region shows the peak from Gly11, which has an extreme ^1^H chemical shift at ~ 4.55 ppm. Residues belonging to the purification tag are labeled in parentheses. **b** Enlargements of the overlapped regions indicated with boxes in panel a showing the assignments in greater detail

The assigned ^1^H–^15^ N HSQC spectrum of ^15^ N/^13^C labled Miro2-nGTPase (residues 1–180) is shown in Fig. 2a. Residues belonging to the purification tag are labeled in parenthesis, otherwise numbering of residues corresponds to the Miro2 sequence. The spectrum exhibits good peak dispersion, indicating that it is well-folded in solution. The peak corresponding to Gly11, located in the G1 motif involved in nucleotide binding, exhibits an extreme ^1^H chemical shift at 4.54 ppm. In the crystal structure of Miro1 nGTPase, which shares a 73% sequence identity with Miro2, the amide proton of Gly11 packs against the aromatic ring of Trp97 (d 2.9 Å), and this likely accounts for the extreme upfield shift of this residue. These types of interactions have been identified for a large number of proteins as contributing to overall stability (Toth et al. 2001). Additionally, Ala149 has a ^15^ N chemical shift of 135.04. This residue is in the G5 motif and canonically hydrogen bonds with the O6 atom of the guanine ring (Pai et al. 1989), which is also found in the Miro1 structure.

Figure 2b depicts two regions in the spectrum with significant peak overlap. Many peaks in this area belong to either residues from the His_6_ purification tag, the unstructured N-terminal residues (1–4), or the unstructured C-terminal residues (172–180). Data obtained from multiple protonated and deuterated samples allowed for unambiguous assignment of nearly all backbone chemical shifts belonging to the Miro2 nGTPase domain. 176 of 180 non-proline ^1^H–^15^ N correlation peaks (97.8%) were assigned. 177 of 180 ^13^C’ (carbonyl) peaks (98.3%) were assigned. 176 out of 180 ^13^Cβ peaks (97.8%) were assigned, and 177 out of 180 ^13^Cα peaks (98.3%) were assigned. Only 34.2% of ^13^Cγ, ^13^Cδ, and ^13^Cε could be assigned from the CCONH-TOCSY dataset. The assignment data has been deposited into BMRB with accession number 51500.

### Prediction of secondary structure

**Fig. 3:**
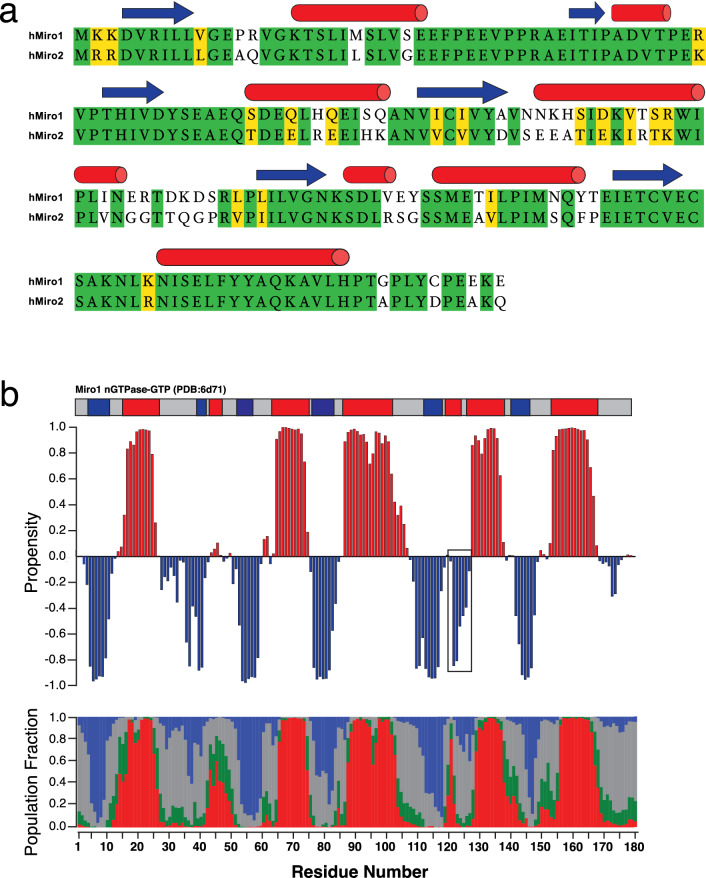
Comparison of Miro2 with Miro1 **a** Amino acid sequence alignment of hMiro1 and hMiro2. The secondary structure elements observed in the crystal structure of hMiro1-GTP are indicated above. **b** Chemical shift-based secondary structure prediction of the Miro2 nGTPase bound to GTP using TALOS-N (upper panel) and CheSPI (lower panel). For TALOS probabilities for helices are indicated in red and extended structure in blue. For CheSPI the relative contributions for the four major types of structure to the chemical shifts are presented as a stacked plot with helix indicated in red, extended(blue), turn (green), and non-structured (grey) The bar above the chart depicts the known secondary structure elements in the deposited structure of Miro1 nGTPase bound to GTP (PDB: 6d71) Helix in red and extended structure indicated in blue)

The secondary structure of Miro2 was predicted using the assigned ^1^H, ^15^ N, ^13^C’, ^13^Cβ, and ^13^Cα chemical shifts using both TALOS-N (Shen and Bax 2013) and CheSPI (Nielsen and Mulder 2021). The results of these two programs are in generally good agreement. The advantage of CheSPI is that it can provide insight into the relative structure and dynamics and can discern the relative contributions of different types of structures that contribute to the chemical shifts. Further, CheSPI can discriminate up to eight types of secondary structure elements for structured proteins. We compared the predicted structure to that of the nGTPase of Miro1 (Smith et al. 2020) (PDB 6d71). Figure 3a shows the amino acid sequence alignment of the nGTPase of human Miro1 and human Miro2 which have 72.78% identity. Figure 3b shows that the structure predictions using TALOS-N and CheSPI closely mirror that seen in the structure of Miro1 nGTPase bound to GTP (represented in the bar above chart). The CheSPI result in Fig. 3b shows the relative contributions from the four major types of structure: helix (red), extended (blue), turn (green) and unstructured (grey). A significant deviation in the prediction for Miro2 compared to Miro1 is seen for residues 122 to 124, which adopt a helical structure in Miro1 but are predicted to adopt a more extended conformation in Miro2. Inspection of the crystal structure of Miro1 reveals that this region forms crystal contacts with a symmetry-related molecule, so the observed differences could arise from packing artifacts. However, confirmation of these differences will require solution analysis of the Miro1 nGTPase domain. Having the GTP sample stable in solution and the backbone assigned allows for future studies in characterizing the interaction of this important protein with its binding partners.

## Data Availability

The chemical shift assignments of Miro2 nGTPase-GTP have been deposited into BMRB with Accession Number 51500.
